# Early renal dysfunction and fibroblast growth factor-23 in patients with small vessel disease-related stroke

**DOI:** 10.1038/s41598-019-51965-5

**Published:** 2019-10-28

**Authors:** Simon Fandler-Höfler, Christian Enzinger, Markus Kneihsl, Daniela Pinter, Sebastian Eppinger, Barbara Obermayer-Pietsch, Anna Goritschan, Hildegard Hafner-Giessauf, Alexander R. Rosenkranz, Franz Fazekas, Thomas Gattringer

**Affiliations:** 1 Department of Neurology, Medical University of Graz, Graz, Austria; 2 Division of Neuroradiology, Vascular and Interventional Radiology, Department of Radiology, Medical University of Graz, Graz, Austria; 3 Division of Endocrinology and Diabetology, Endocrinology Lab Platform, Department of Internal Medicine, Medical University of Graz, Graz, Austria; 4 Division of Nephrology, Department of Internal Medicine, Medical University of Graz, Graz, Austria

**Keywords:** Chronic kidney disease, Stroke, Stroke

## Abstract

Interactions between cerebral small vessel disease (CSVD) and renal dysfunction (RD) have been reported, but previous studies were mostly retrospective and limited to measurements of estimated glomerular filtration rate (eGFR). In this prospective, longitudinal study of patients with CSVD-related recent small subcortical infarcts (RSSI), we aimed at a comprehensive exploration of markers of early RD and their association with microvascular brain damage. We investigated 101 stroke patients (mean age: 60.2 ± 10.7 years) with an MRI-confirmed RSSI who underwent follow-up brain MRI 15 months post-stroke. Besides serum creatinine and eGFR, we assessed urinary Albumin-Creatinine Ratio and fibroblast growth factor-23 (FGF-23). RD was classified according to recent Kidney Disease: Improving Global Outcomes criteria. We identified 24 patients with RD, only six patients revealed an eGFR <60 mL/min/1.73 m². RSSI patients with RD more often had severe white matter hyperintensities (WMH, 58% vs. 36%, p = 0.04). CSVD progression was not dependent on RD. However, patients in the highest FGF-23 quartile more frequently had new microangiopathic lesions on follow-up MRI (50% vs. 21%, p = 0.03). Early RD was found in a quarter of RSSI patients and associated with WMH severity, but not CSVD progression. High FGF-23 indicates an increased risk for ongoing microvascular brain damage, warranting further studies.

## Introduction

Approximately a quarter of all ischemic strokes are caused by the occlusion of a small perforating brain artery, formerly termed lacunar stroke, and related to cerebral small vessel disease (CSVD). The neuroimaging correlate is a recent small subcortical infarct (RSSI) and most sensitively diagnosed using diffusion-weighted MRI^1^. Aside from RSSI, chronic neuroimaging features of CSVD include lacunes as residues of such infarcts, white matter hyperintensities (WMH), microbleeds and enlarged perivascular spaces^1^.

The pathophysiology of CSVD is incompletely understood. It has been suggested that CSVD may be part of a multisystemic small vessel disease also affecting other vascular beds such as the retina or kidneys^2^. Especially small vessels of both brain and kidneys share anatomical and functional characteristics, which likely makes them vulnerable to similar risk factors and pathophysiological mechanisms. Shared functional characteristics include requirement of constant high blood flow through a low-resistance vascular system and autoregulation^3,4^.

While previous investigations consistently showed a higher burden of CSVD-related brain damage (WMH, lacunes, microbleeds and enlarged perivascular spaces) in lacunar stroke patients with renal impairment^5,6^, a meta-analysis found no difference in the incidence of renal dysfunction (RD) between lacunar versus non-lacunar stroke patients^7^. However, most studies incorporated in this analysis identified lacunar stroke on the basis of clinical symptomatology, i.e. the presence of a lacunar syndrome, which is rather unreliable compared to radiological classification with diffusion-weighted MRI^8^. Also, the majority of studies which explored the association of stroke with RD were retrospective and used the estimated glomerular filtration rate (eGFR) as the sole parameter of RD, although current definitions of chronic kidney disease (CKD) demand the inclusion of albuminuria^9^.

Fibroblast growth factor 23 (FGF-23) is a cell signalling protein that has recently been identified as an early marker of RD^10^. Moreover, elevated FGF-23 levels have been associated with higher rates of cardiovascular events in patients with RD^11,12^ and in population-based studies^13^. Therefore, FGF-23 could be an interesting biomarker in patients with CSVD-related stroke and might be related to their prognosis.

We therefore planned a comprehensive assessment of renal dysfunction (including eGFR, albuminuria and FGF-23) in a prospective cohort of MRI-defined RSSI patients who also underwent control brain MRI at 15-months after stroke. We hypothesized that the incorporation of albuminuria to the definition of RD would increase detection of patients at increased risk for more severe CSVD-related brain changes at baseline and might relate to the progression of CSVD at follow-up. Secondly, we particularly explored the value of FGF-23 as a potential predictor of ongoing CSVD-related brain damage.

## Methods

### Study participants

From May 2012 to December 2016, all stroke patients ≤75 years with an MRI-confirmed RSSI were invited to participate in this prospective study which has in part already been reported elsewhere^14^. Exclusion criteria included acute brain infarcts of other aetiologies, pre-stroke modified Rankin Scale >1 and contraindications for MRI (e.g. metal implants or claustrophobia). We asked especially for any vascular events during the follow-up period and confirmed those by medical records.

### Stroke work-up and neuroimaging

At the time of stroke (baseline), patients underwent a thorough neurological examination and workup including brain MRI, electrocardiography (ECG), duplex sonography of brain-supplying vessels, 24h-ECG, echocardiography as well as blood and 24-hour urine sampling. In-hospital follow-ups at the stroke outpatient department were carried out at three and 15 months after stroke and included a neurological examination, MRI of the brain and blood sampling.

At baseline, all study patients underwent brain MRI at 1.5 Tesla (Siemens MAGNETOM Espree, Siemens Healthcare, Erlangen, Germany) according to a standard protocol for the workup of patients with suspected cerebrovascular events^14^. At both follow-ups, brain MRI was performed on a 3 Tesla TimTrio or Prisma scanner (Siemens Healthcare, Erlangen, Germany). All MRI scans were reviewed by a neuroradiological expert (CE) according to the STandards for ReportIng Vascular changes on nEuroimaging (STRIVE)^1^, blinded to clinical data. Follow-up MRI scans were specifically analysed with regard to changes in cerebrovascular lesions (such as new infarcts, lacunes and WMH progression) as previously described^14,15^. More specifically, WMH were rated according to the Fazekas scale^16,17^. Progression of WMH was defined the as occurrence of new white matter hyperintensities or transition to a higher Fazekas scale score (punctate foci to early-confluent lesions, early-confluent lesions to large confluent areas)^18^.

### Renal parameters

We determined creatinine and the eGFR was calculated using the CKD-EPI creatinine equation^19^. We also analysed albuminuria and the urinary Albumin-Creatinine Ratio (uACR, mg albumin/g creatinine). The uACR has been recommended as the most reliable marker of albuminuria because of known problems with 24-hour urine albuminuria (including problems in proper collection and difficulties in correction towards sex, age, weight, and other parameters)^9,20^. Therefore, we chose the uACR calculated from the 24-hour urine sample as our main marker of albuminuria. Investigated blood parameters at baseline further included C-terminal FGF-23, which was measured by a 2nd generation enzyme-linked immune-sorbent assay (ELISA) by Immuntopics Inc., San Clemente, CA. Sensitivity of the assay was 1.5 RU/mL, coefficient of intra-assay variation (20 duplicate measurements of two samples) was detected as 1.4 to 2.4%, inter-assay variation (duplicate determinations of 2 samples performed in 10 assays) was 2.4 to 4.7%. Peripheral blood was taken by venepuncture together with the collection of urine samples (mean time from stroke symptom onset to blood sampling: 5.2 ± 2.7 days).

We defined RD/CKD according to the proposed Kidney Disease: Improving Global Outcomes Clinical Practice (KDIGO) guideline (either eGFR <60 mL/min/1.73 m² and/or albuminuria/ACR >30 mg/g). High-risk RD patients were defined as eGFR <45 mL/min/1.73 m², eGFR <60 mL/min/1.73 m² with albuminuria/ACR >30 mg/g or any GFR with albuminuria/ACR >300 mg/g^9^.

At follow-up examinations, we analysed serum creatinine, and the respective eGFR, as well as FGF-23 from peripheral blood.

### Data analysis

For data analysis, we first explored the association of RD with traditional vascular risk factors and the extent of CSVD-related brain damage at baseline in a cross-sectional manner. In a second step, we searched for associations between MRI-confirmed CSVD progression at follow-up with renal parameters both at baseline and follow-up.

Progression of CSVD was defined either as a recurrent RSSI, or the detection of a new lacunes or new WMH on the 15-months-follow-up MRI.

### Statistical analysis

Statistical analysis was performed using IBM SPSS Statistics 22. Continuous variables were evaluated for normal distribution using the Kolmogorov-Smirnov test. Normally distributed continuous variables were compared by the unpaired Student’s t-test, for other distributions the Mann-Whitney U-test was used. For categorical variables, we used the chi-square test and univariate logistic regression. P-values of equal or less than 0.05 were considered statistically significant.

The study was approved by the ethics committee of the Medical University of Graz (ethics committee number: 24–260 ex 11/12) and was carried out in accordance with the relevant guidelines and regulations. All patients gave written informed consent.

## Results

### Characteristics of the cohort

We identified 101 RSSI patients who agreed to participate in the study period (mean age 60.2 ± 10.7 years, 73% men). Of those, 98 (97%) completed the 15-month follow-up, while three patients only completed the follow-up investigation at three months. In those three patients, data from their three-month follow-up was used in the prospective analysis.

Baseline characteristics including clinical information, vascular risk factors, laboratory and MRI findings are shown in Table 1.

**Table 1:** Cross-sectional analysis of the study cohort regarding renal dysfunction.

	Study Cohort	Patients with RD	Patients without RD	p-value
	n = 101	n = 24 (23.8%)	n = 77 (76.2%)	
**Clinical features**				
Age, years (mean ± SD)	60.2 ± 10.7	63.3 ± 9.6	59.3 ± 10.9	0.11
Women, n (%)	27 (26.7%)	7 (29.2%)	20 (26.0%)	0.76
Arterial hypertension, n (%)	77 (76.2%)	20 (83.3%)	57 (74.0%)	0.35
Dyslipidemia, n (%)	80 (79.2%)	23 (95.8%)	57 (74.0%)	**0.02**
Diabetes mellitus, n (%)	16 (15.8%)	8 (33.3%)	8 (10.4%)	**0.01**
Smoker (ever), n (%)	42 (41.6%)	11 (45.8%)	31 (40.3%)	0.63
Obesity, n (%)	19 (18.8%)	6 (25.0%)	13 (16.9%)	0.37
Atrial fibrillation, n (%)	4 (4%)	1 (4.2%)	3 (3.9%)	0.95
Coronary heart disease, n (%)	7 (6.9%)	3 (12.5%)	4 (5.2%)	0.22
History of stroke, n (%)	4 (4%)	2 (8.3%)	2 (2.6%)	0.21
NIHSS at admission, median (range)	2 (0–9)	3 (1–6)	2 (0–9)	0.33
**Laboratory findings at baseline**				
eGFR (mL/min/1.73 m²)	80.5 ± 15.4	72.0 ± 18.6	83.2 ± 13.4	**0.001**
ACR >30 mg/g, n (%)*	22 (22.9%)	22 (95.7%)	0	<**0.001**
FGF-23 (rU/mL)	96.0 ± 58.3	111.4 ± 75.8	91.1 ± 51.2	0.29
FGF-23, 1^st^ quartile (<62 rU/mL)	26 (26.0%)	5 (20.8%)	21 (27.6%)	0.51
FGF-23, 4^th^ quartile (>112 rU/mL)	24 (24.0%)	9 (37.5%)	15 (19.7%)	0.08
**MRI findings at baseline**				
RSSI size (mm axial, mean ± SD)	11.8 ± 4.3	12.2 ± 4.7	11.6 ± 4.2	0.58
WMH Fazekas score 2–3, n (%)	42 (41.6%)	14 (58.3%)	28 (36.4%)	**0.04**
Lacunes, n (%)	36 (35.7%)	11 (45.8%)	25 (32.5%)	0.23
Microbleeds, n (%)	14 (13.9%)	3 (12.5%)	11 (14.3%)	0.83
Old cortical infarcts, n (%)	8 (7.9%)	4 (16.7%)	4 (5.2%)	0.07

ACR: Albumin-Creatinine Ratio; CSVD: Cerebral small vessel disease; eGFR: Estimated glomerular filtration rate; FGF-23: Fibroblast growth factor 23, NIHSS: National Institutes of Health Stroke Severity Scale; mRS: Modified Rankin Scale; RD: Renal dysfunction, WMH: White matter hyperintensities. *missing in five patients.

### Renal dysfunction

Average eGFR at baseline was 80.5 ± 15.4 mL/min/1.73 m². Only six patients (5.9%) had an eGFR <60 mL/min/1.73 m², while 22 patients (22.9%) had a pathological uACR. According to the proposed KDIGO criteria, 24 patients (23.8%) thus had RD, and six patients (5.9%) had high-risk RD (Table 1**)**.

### Cross-sectional analysis of patients with renal dysfunction

RSSI patients with RD more frequently had diabetes mellitus (33.3% vs. 10.4%) and dyslipidaemia (95.8% vs. 74.0%). All other risk factors were equally distributed among patients with versus without RD (Table 1**)**.

Severe WMH (i.e. early confluent and confluent WMH according to Fazekas scale scores 2 and 3) were more often present in patients with versus without RD (58.4% vs. 36.4%, p = 0.04). The distribution of other neuroimaging markers of CSVD was not different (Table 1**)**. Patients with severe WMH at baseline also had significantly lower eGFR both at baseline (75.6 ± 13.5 vs. 84.0 ± 15.9 mL/min/1.73 m², p < 0.01) and at follow-up (72.9 ± 13.9 vs. 81.7 ± 16.8 mL/min/1.73 m², p < 0.01). FGF-23 levels were not related to WMH severity or the presence of lacunes or microbleeds on brain MRI at baseline (data not shown).

### Progression of CSVD

During the 15-months-study period, 12 patients (11.9%) had progression of CSVD (two had a recurrent symptomatic RSSI, seven showed new silent lacunes and three others had new WMH on MRI). Classical vascular risk factors did not predict CSVD progression (Table 2**)**. Patients with severe WMH, microbleeds but also with old cortical infarcts on baseline MRI more frequently had CSVD progression **(**Table 2**)**.

**Table 2:** Clinical, laboratory and radiological parameters in patients with versus without progression of CSVD.

	Study Cohort	CSVD Progression	No CSVD Progression	p-value
	n = 101	n = 12 (11.9%)	n = 89 (88.1%)	
**Clinical features at baseline**				
Age, years (mean ± SD)	60.2 ± 10.7	62.1 ± 8.4	60.0 ± 11.0	0.53
Women, n (%)	27 (26.7%)	4 (33.3%)	23 (25.8%)	0.58
Arterial hypertension, n (%)	77 (76.2%)	11 (91.7%)	66 (74.2%)	0.18
Dyslipidemia, n (%)	80 (79.2%)	9 (75.0%)	71 (79.8%)	0.70
Diabetes mellitus, n (%)	16 (15.8%)	1 (8.3%)	15 (16.9%)	0.45
Smoker (ever), n (%)	42 (41.6%)	5 (41.7%)	37 (41.6%)	0.99
Obesity, n (%)	19 (18.8%)	3 (25.0%)	16 (16.0%)	0.56
Atrial fibrillation, n (%)	4 (4%)	0 (0%)	4 (4.5%)	0.45
Coronary heart disease, n (%)	7 (6.9%)	0 (0%)	7 (7.9%)	0.31
History of stroke, n (%)	4 (4%)	1 (8.3%)	3 (3.4%)	0.41
NIHSS at admission, median (range)	2 (0–9)	2 (0–4)	2 (0–9)	0.23
**Laboratory findings at baseline**				
eGFR (mL/min/1.73 m²)	80.5 ± 15.4	75.1 ± 19.7	81.3 ± 14.8	0.20
eGFR <60 (mL/min/1.73 m²)	6 (5.9%)	1 (8.3%)	5 (5.6%)	0.71
eGFR 60–89 (mL/min/1.73 m²)	64 (63.4%)	9 (75.0%)	55 (61.8%)	0.37
eGFR ≥ 90 (mL/min/1.73 m²)	31 (30.7%)	2 (16.7%)	29 (32.6%)	0.26
ACR >30 (mg/g)	22 (22.9%)	4 (33.3%)	18 (21.4%)	0.36
ACR >300 (mg/g)	5 (5.2%)	1 (8.3%)	4 (4.8%)	0.60
Renal dysfunction, n (%)	24 (23.8%)	4 (33.3%)	20 (22.5%)	0.41
High-risk renal dysfunction, n (%)	6 (5.9%)	1 (8.3%)	5 (5.6%)	0.54
FGF-23, 1^st^ quartile (<62 rU/mL)	26 (26.0%)	2 (16.7%)	24 (27.3%)	0.43
FGF-23, 4^th^ quartile (>112 rU/mL)	24 (24.0%)	6 (50.0%)	18 (20.5%)	**0.03**
FGF-23 (rU/mL)	92.4 ± 59.1	104.1 ± 39.9	94.9 ± 60.5	0.61
**MRI findings at baseline**				
RSSI size (mm axial, mean ± SD)	11.8 ± 4.3	12.3 ± 3.8	11.7 ± 4.4	0.67
WMH Fazekas score 2–3, n (%)	42 (41.6%)	9 (75.0%)	33 (37.1%)	**0.01**
Lacunes, n (%)	36 (35.7%)	7 (58.3%)	29 (32.6%)	0.08
Microbleeds, n (%)	14 (13.9%)	5 (41.7%)	9 (10.1%)	**0.003**
Old cortical infarcts, n (%)	8 (7.9%)	3 (25.0%)	5 (5.6%)	**0.02**
Laboratory findings at 15 months*				
eGFR (mL/min/1.73 m²)	78.0 ± 16.2	70.8 ± 15.5	79.0 ± 19.5	0.10
eGFR <60 (mL/min/1.73 m²)	10 (9.9%)	3 (25.0%)	7 (7.9%)	0.06
FGF-23 (rU/mL)	107.9 ± 77.9	133.3 ± 58.7	104.4 ± 79.8	0.23
FGF-23, 1^st^ quartile (<64 rU/mL)	25 (25.0%)	1 (8.3%)	24 (27.3%)	0.16
FGF-23, 4^th^ quartile (>127 rU/mL)	25 (25.0%)	6 (50.0%)	19 (21.6%)	**0.03**

ACR: Albumin-Creatinine Ratio; CSVD: Cerebral small vessel disease; eGFR: Estimated glomerular filtration rate; FGF-23: Fibroblast growth factor 23, NIHSS: National Institutes of Health Stroke Severity Scale; mRS: Modified Rankin Scale; WMH: White matter hyperintensities; *15 months follow-up was not available in three patients, in those, data from the 3-months follow-up were analysed.

Patients with RD (as measured by eGFR, uACR and RD risk categories) did not more frequently show progression of CSVD. Similarly, absolute FGF-23 levels were not different between patients with versus without CSVD progression. However, patients in the highest FGF-23 quartile (>112 RU/mL, n = 24) at baseline compared to all other patients more frequently had new CSVD-related ischemic brain changes at follow-up (p = 0.03, OR 3.89). These results were similar when analysing the highest FGF-23 quartile at follow-up (p = 0.03, OR 3.63, Table 2) and remained significant after correction for age and sex (baseline: p = 0.03, follow-up: p = 0.04).

**Figure 1:**
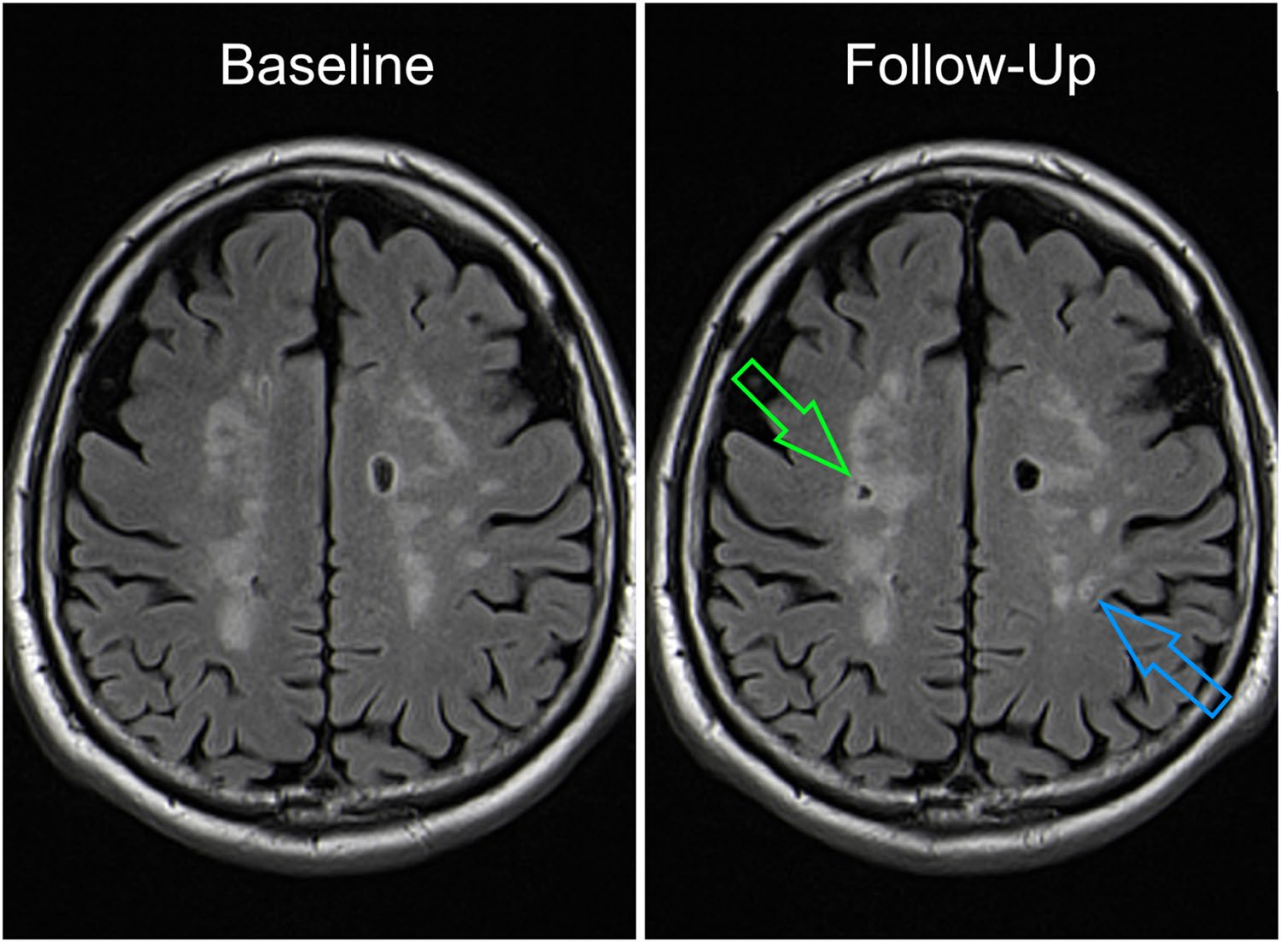
Brain MRI (FLAIR sequence) of a 72-year-old male patient with progression of cerebral small vessel disease during the observation period. The left image shows the baseline MRI (recent infarct not shown), the right image the follow-up MRI after fifteen months depicting a new lacune in the right centrum semiovale (green arrow) and a new white matter hyperintensity in the left centrum semiovale (blue arrow), which occurred clinically asymptomatic. Aside from well-controlled arterial hypertension, this patient had no further vascular risk factors. Baseline eGFR was 68 ml/min/1.73 m², the patient had no increased albuminuria/ACR, but FGF-23 was increased at 159 rU/mL. Follow-up laboratory assessments showed decreasing eGFR (45 ml/min/1.73 m² at fifteen months) and increasing FGF-23 (168 rU/mL at fifteen months).

**Figure 2:**
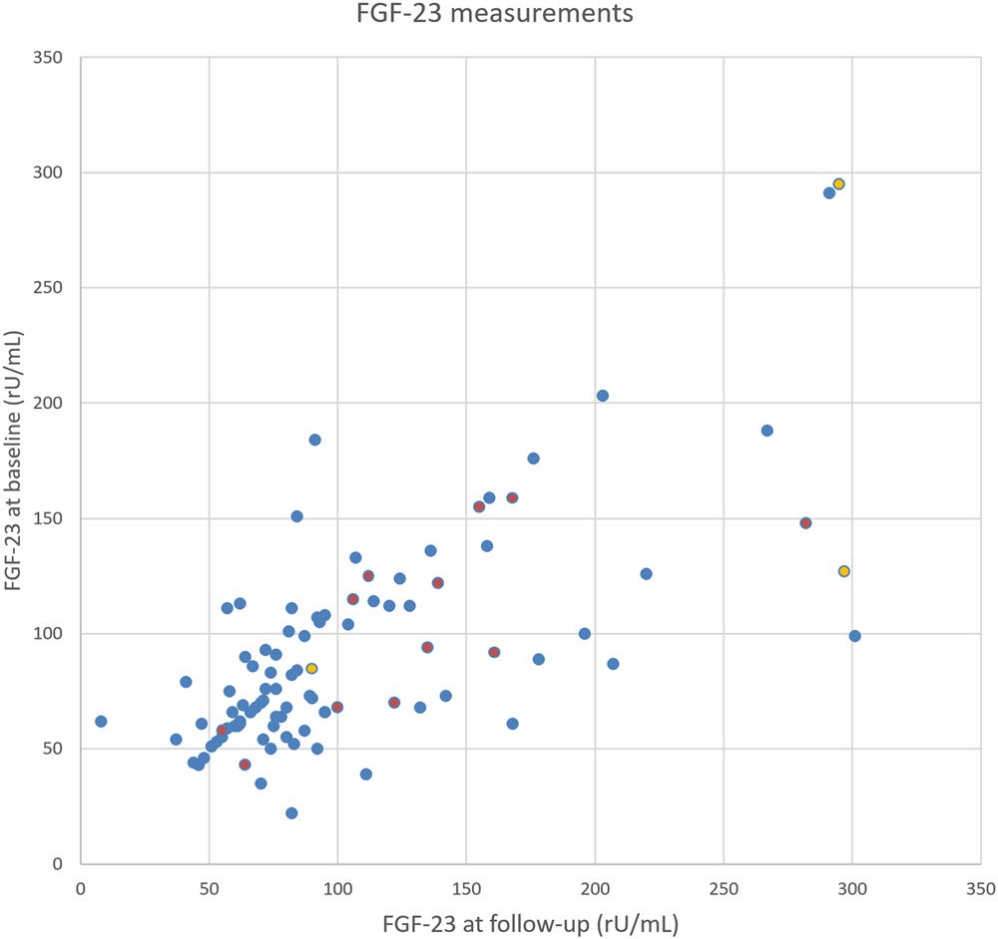
Scatter plot of FGF-23 levels at baseline and at follow-up (one point represents one patient). Individual patients with progression of cerebral small vessel disease are shown in red, patients with other cardiovascular events during the observational period are highlighted in yellow.

An example of a patient with a high FGF-23 level and CSVD progression is shown in Figure 1. Figure 2 illustrates individual FGF-23 levels at baseline and follow-up, highlighting patients with CSVD progression and recurrent vascular events.

### Recurrent vascular events

During the 15-months observation period, three non-CSVD-related vascular events occurred (one macroangiopathy-related cortical ischaemic infarct, one cortical infarct in the posterior cerebral artery territory and one myocardial infarction).

If we extended the group with CSVD progression by those patients with recurrent vascular events, that group more frequently had high-risk RD (20% vs. 3.5%, OR 6.92, p = 0.01) and more often belonged to the highest FGF-23 quartile (53.3% vs. 18.8%, OR 4.93, p = 0.003), while traditional vascular risk factors were not different.

### Progression of renal dysfunction

Overall, 23 patients (22.8%) developed a significant decrease of renal function (GFR reduction by more than 10 mL/min/1.73 m²) at the 15-month-follow-up. While not statistically significant, patients with worsening renal function more often had CSVD progression (21.7 vs. 9.0%, OR 2.82, p = 0.10) or any vascular endpoint event (26.1 vs. 11.5%, OR 2.71, p = 0.09). Also, those patients more frequently were in the highest FGF-23 quartile at follow-up (36.0% vs. 17.3%, OR 2.68, p = 0.05) and tended to be in the highest FGF-23 quartile at baseline (33.3% vs. 18.4%, OR 2.21, p = 0.12).

## Discussion

In our series of patients aged ≤75 years with an MRI-confirmed RSSI, about one quarter had evidence for early stage renal dysfunction according to the recently proposed KDIGO criteria. This was associated with an increased frequency of diabetes mellitus and dyslipidaemia, and patients with RD more often had early confluent to confluent WMH. Progression of CSVD as visible on MRI during a follow-up period of 15 months was not related to conventional parameters of RD; however, higher FGF-23 levels were associated with accumulating microangiopathic brain damage within the follow-up period as well as with vascular events in general.

Extending previous work, which mainly assessed eGFR, we here applied the recently proposed KDIGO definition of RD/CKD by integrating both eGFR and albuminuria. This helps identifying patients with early stage RD - a subgroup that would particularly profit from preventive strategies^21^. While only six patients in our study cohort had an eGFR <60 mL/min/1.73 m², 22 patients had a pathological uACR, resulting in 24 patients with mainly mild RD. The cross-sectional analysis at baseline revealed that even those patients with mild RD already more often had severe WMH. These results are in line with a study of lacunar stroke patients showing a higher total burden of CSVD in patients with RD and an investigation of young stroke patients which reported a lower eGFR in patients with more severe WMH^5,22^. A larger recent study investigating patients with different stroke subtypes including transient ischemic attacks also corroborates these findings in that WMH were significantly more severe in patients with RD younger than 60 years, which was the mean patient age in our study^23^.

The association of severe WMH and RD could be a consequence of exposure to same vascular risk factors known to affect the cerebral and renal microvasculature (e.g. lifetime hypertension)^4^. Hypertension was also highly prevalent in our patients but not to a significantly different extent between patients with and without RD, while we found such differences for diabetes mellitus and dyslipidaemia. Conversely, a number of direct mechanisms acting on both organs have also been suggested, including nitric oxide deficiency in RD^24^, hyperphosphatemia and associated arterial calcification^25^, deficiency of calcification inhibitors like Klotho, a co-receptor for FGF-23^26^, and others^3^. The presence of lacunes and microbleeds were not associated with RD in our study. This may be a consequence of mild RD and a weaker association between RD and those two CSVD markers than with WMH as shown in a previous study^23^. As usual, lacunes and microbleeds also occurred more rarely in our patients in general which may have prohibited adequate statistical assessment. We did not investigate enlarged perivascular spaces, which have been shown to be associated with markers of RD in a previous study^5^.

CSVD progression was observed especially in patients with severe WMH, microbleeds and old cortical infarcts, which is in line with earlier observations of this association^27^. Interestingly, CSVD progression at 15 months was not different when comparing patients with and without RD or when analysing the various conventional parameters of RD separately. Again, this is likely a consequence that RD was mostly mild in our patients. However, elevated FGF-23 was associated with the occurrence of new CSVD-related brain changes in longitudinal analysis. Patients within the highest FGF-23 quartile also experienced an overall higher proportion of vascular events and accumulating cerebral ischemic damage.

Previous studies have shown a higher occurrence of recurrent cardiovascular events in the highest quartile of FGF-23 in patients with acute coronary syndrome as well as worse outcomes in patients with known RD, including increased frequency of heart failure and mortality^11,12,28^. A population-based study of stroke-free individuals found that FGF-23 is a risk factor for subsequent stroke independent of RD^29^, an association in CSVD with progressing vascular disease has not yet been reported. FGF-23 concentrations in our study (median 76 RU/ml) were higher compared to population-based cohorts (median 43–74 RU/ml) but lower compared to studies on patients with CKD (median 102–392 RU/ml). Most previous investigations on FGF-23 were therefore performed using quartiles for comparison as we have done^13^. To the best of our knowledge, this is the first investigation of FGF-23 in patients with CSVD. Our finding of a worse disease course in RSSI patients with high FGF-23 levels demands additional investigations, especially with regard to potential cut-off values for indicating an increased risk for progressive (micro)vascular changes.

Strengths of our study include the precise definition of renal dysfunction according to recently proposed criteria also incorporating early disease stages as well as the MRI-based definition of RSSI and CSVD according to current guidelines^9^. Furthermore, repeated MRI at prospectively defined follow-up investigations allowed for exact identification of neuroimaging markers of CSVD and their progress. However, our study also has some relevant limitations. These come mainly from the relatively small patient number with few outcome events which prohibited accounting for covariates in multivariate analysis. Therefore, the findings of our study need to be regarded as exploratory and hypothesis-generating. As our study design aimed for very high follow-up completion rates and to minimize the influence of concomitant neurodegenerative brain changes, we decided to exclude patients above 75 years of age. This also explains why our study cohort contained primarily mildly affected patients, as vascular risk factors, RD and the incidence of cardiovascular events are strongly affected by age^30^. On the other hand this could be viewed as even strengthening the impact of our observations which thus were made already in the presence of mostly mild RD and FGF-23 elevations. Studies of larger, unselected study cohorts with longer follow-up periods using volumetric WMH quantification tools in comparison with well-established visual WMH progression scales^18,27,31^ will now be needed for confirmation.

## Data Availability

The datasets generated and/or analysed during this study are not publicly available, but are available from the corresponding author on reasonable request.
